# Role of platelet indices as a biomarker for the diagnosis of acute appendicitis and as a predictor of complicated appendicitis: A meta-analysis

**DOI:** 10.1016/j.amsu.2021.102448

**Published:** 2021-05-27

**Authors:** Thawatchai Tullavardhana, Sarat Sanguanlosit, Anuwat Chartkitchareon

**Affiliations:** Department of Surgery, Faculty of Medicine, Srinakharinwirot University, Ongkharak, Nakhon Nayok, 26120 , Thailand

**Keywords:** Acute appendicitis, Platelet indices, Mean platelet volume

## Abstract

**Introduction:**

Acute appendicitis is one of the most common surgical emergencies worldwide. Clinical scoring system systems have been developed to diagnose acute appendicitis, but insufficient to predict the complication. The amount of serum biomarkers elevates in response to acute inflammation, which could be beneficial for diagnostic tools. Accordingly, a meta-analysis was conducted to evaluate the efficacy of platelet indices, including mean platelet volume (MVP) and platelet distribution width (PDW) as potential biomarkers for the diagnosis of a diagnosis of acute appendicitis.

**Material and methods:**

The dataset was defined by searching for articles published until December 2020 from PubMed, EMBASE, Google Scholar and the Cochrane database. The meta-analysis was performed using Review Manager Software version 5.4.1.

**Results:**

The final analysis was made from 9 studies, including 3124 patients. The results demonstrated that lower MPV values was significantly associated with acute appendicitis (odds ratio (OR) = 0.81, 95% confidence interval (CI) = −1.51 to −0.11, P = 0.02), but not associated with complicated appendicitis by comparing it with the control (OR = −0.13,95% CI = −0.33 to −0.07, P = 0.19) and non-complicated appendicitis groups (OR = −0.13,95% CI = −0.30 to −0.04, P = 0.14). The present study failed to demonstrate the diagnostic value of PDW for the prediction of appendicitis and its complication.

**Conclusion:**

The results of the meta-analysis strongly indicate that a lower MVP values could function as a marker for predicting the acute appendicitis.

## Highlights

• Platelet play an important role in the response to systemic inflammation and sepsis.
• Inflammatory process activated platelets destruction consequence in the release of small platelets into bloodstream.
• This studycompared the association between the platelet indices and the diagnosis of acute appendicitis.
• Lower mean platelet volume(MVP) is a significant diagnostic marker for acute appendicitis.

## 1 Introduction

Acute appendicitis (AA) is one of the most common surgical emergencies worldwide. The annual incidence of acute appendicitis is 9.4 per 10,000 people and is continuing to increase in newly industrialized countries [1,2]. Urgent appendectomy within 24 h remains a part of standard care for patients with acute appendicitis. Diagnosis and treatment delays may be associated with the development of complicated appendicitis, which increases postoperative complications and 30-day mortality by up to 8% and 0.6%, respectively [3,4].

Several clinical scoring systems have been developed to diagnose acute appendicitis with greater than 80% sensitivity and specificity among high-risk patients, such as the Alvarado score, Appendicitis Inflammatory Response (AIR) score, and RIPASA (Raja Isteri Pengiran Anak Saleha Appendicitis) score. Nevertheless, the clinical scoring system insufficiently diagnoses acute appendicitis and appears to be unreliable at differentiating between complicated and uncomplicated disease [[5], [6], [7]].

Radiological methods include ultrasonography and computed tomography (CT) scans which have become valuable diagnostic tools. Point of care ultrasound (POCUS) is preferable for initial investigations and has a sensitivity and specificity for diagnosing AA of 91% and 97%, respectively [8]. The low dose CT scan is effective as a standard dose in diagnosing acute appendicitis with a sensitivity of 94% and 95%, respectively. Hence, these methods are useful for the diagnosis of complicated appendicitis [9]. However, the major limitations of these examinations are their high cost and lack of availability in the majority of rural hospitals in developing countries.

Complete blood count (CBC) is one of the most common basic laboratory tests to determine inflammatory pathology. Leukocytosis and the neutrophils shift to the left are associated with acute appendicitis. The amount of several biomarkers elevate in response to acute inflammation, including C-reactive, Neutrophil-Lymphocyte ratio (NL-ratio), and Platelet-Lymphocyte ratio making them reliable tools in the identification of non-complicated and complicated appendicitis [[10], [11], [12]].

Platelet indices play an important role in the response to systemic inflammation and sepsis [13]. High inflammation activity increases the activation, sequestration, and destruction of platelets which results in the release of small platelets into the bloodstream [14]. Platelet indices include mean platelet volume (MVP) and platelet distribution width (PDW) which is a measurement of platelet size determined and determined through a routine complete blood count test. Therefore, the reduction of MPV and PDW parameters may be used as a marker to reflect the acute inflammation burden and disease severity for several diseases, including rheumatoid arthritis acute pancreatitis and inflammatory bowel disease [15,16].

Although several studies report the usefulness of platelet indices in the diagnosis of acute appendicitis, there has been a significant degree of inconsistency in the findings of these studies. The present study conducts a meta-analysis based on the published literature in an attempt to clarify and evaluate the efficacy of platelet indices (MPV and PDW) as a potential biomarker for a diagnostic test of acute appendicitis and complications.

## 2 Material and methods

### 2.1 Data sources and search strategies

Electronic literature searches were performed on PubMed, Embase, Google Scholar, and the Cochrane database. The search terms ‘platelet indices’, ‘biomarkers’, ‘acute appendicitis’, and ‘complicated appendicitis’ were used as keywords to identify all English-language studies published through to December 2020 that evaluated the role of platelet indices (MPV and PDW) as a biomarker in the diagnosis of acute appendicitis and in the prediction of complicated appendicitis. Meta-analysis was performed according to the PRISMA 2020 statement, a guideline for reporting systematic reviews [17]. The quality of the meta-analysis was assessed using the AMSTAR 2 criteria critical appraisal tool for systematic reviews that include randomized or non-randomized studies of healthcare interventions [18]. The protocol of this meta-analysis was registered on Review Registry (Unique Identifying Number of reviewregistry1131) [19].

### 2.2 Study selection and eligibility criteria

The study inclusion criteria were as follows: (1) Studies published in English; (2) Acute appendicitis proven by the pathologic diagnosis; (3) Clarification of complicated appendicitis as gangrenous or perforated appendicitis; (4) Clarification that the control group were patients with non-diagnosis of acute appendicitis or normal appendix through operative or pathological findings; and (5) Measurement of platelet indices to compare between control and non-complicated or complicated appendicitis groups. The exclusion criteria were: (1) Non-English language articles; (2) review articles; (3) studies involving pediatric patients (aged < 15 years); and (4) no continuous variable data reported. The quality of the studies included in the meta-analysis was further evaluated using the Newcastle-Ottawa scale. The maximum possible score is 9 points which represents the highest methodological quality [20].

### 2.3 Statistical analysis

The two reviewers independently extracted the following information from the selected studies: Author names, country of origin, year of publication, study design, number of patients, patient characteristics, levels of MPV and PDW, and clinicopathologic diagnosis of acute appendicitis. Extracted data was cross-checked to reach consensus and then entered into a computerized spreadsheet for analysis.

Meta-analysis was performed using Review Manager software, version 5.4.1 which was provided by the Cochrane Collaboration (Nordic Cochrane Center, Cochrane Collaboration, Copenhagen, Denmark). Cochrane's chi-square-based Q-statistic test was applied to assess between-study heterogeneity. An I^2^ statistic was used to test for heterogeneity between the included studies (p < 0.05 is considered for significant heterogeneity).

The association between platelet indices (MPV and PDW) with the diagnosis acute appendicitis and the prediction of complicated appendicitis by comparing with the control group was analyzed using continuous variable data (mean ± SD) with inverse variant methods to generate a pooled odds ratio (OR). The OR was considered statistically significant at the p < 0.05 level and the 95% confidential interval (CI) did not include the value 1.

The authors adopted the random-effects model, which is a more conservative way to calculate ORs, assumes a high level of variety between studies, and uses a weighted average of the effects reported in different studies to calculate levels of association. Publication bias was assessed by visual examination of a funnel plot, while asymmetry was formally assessed using both Egger's linear regression test and the rank correlation test (Begg's test).

## 3 Results

**Fig. 1:**
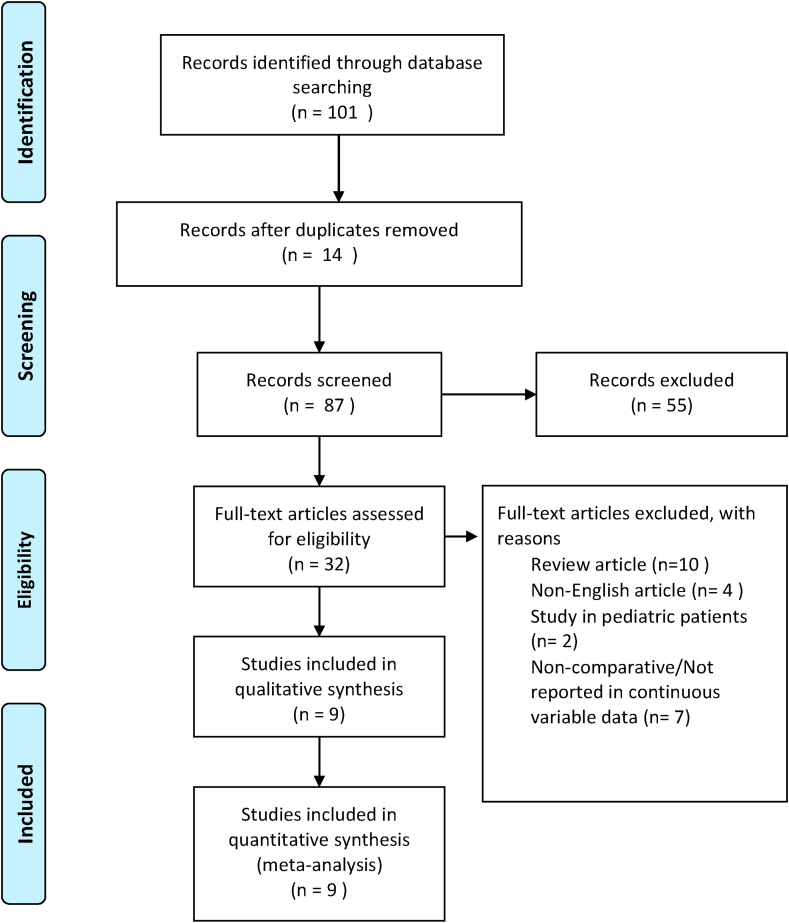
Selection process of studies for inclusion in the meta-analysis.

The initial search identified a total of 101 potential articles. After screening, nine articles that matched the researchers’ criteria were deemed suitable for inclusion in the meta-analysis [[21], [22], [23], [24], [25], [26], [27], [28], [29]]. The PRISMA diagram used in the search process is shown in Fig. 1. The two reviewers showed 100% agreement with the final dataset. The pooled studies included 3124 patients which was used to investigate the association between the platelet indices (MPV, PDW) values with the diagnosis of acute appendicitis by comparing acute appendicitis and complicated appendicitis against control groups.

Patients with hematologic disorder and severe comorbidities such as liver disease, heart failure, and peripheral vascular disease, which may affect the levels of MPV and PDW, were excluded from all of the included studies. Blood samples were obtained from the patient's admission time or within 24 h after diagnosis of acute appendicitis and drawn into tubes containing EDTA or citrate to analyze complete blood count and platelet indices using an automated hematologic analyzer. The Newcastle-Ottawa scale found that all the studies included in the meta-analysis were of moderate to good quality (6–8 stars). The characteristics of the nine included studies are shown in Table 1.

**Table 1:** Characteristics of the 9 included studies included in the meta-analysis regarding the platelet indices and acute appendicitis.

Study	Country	Year	Study design	Number of patients	Mean age (years)	Blood sample obtained and analysis	Platelet indices	Matching	Newcastle Ottawa score
Albayrak	Turkey	2011	Retrospective study	432	- Appendicitis group: 32.5 ± 15.1	- At the time of admission	Mean platelet volume (MPV)	a, b, c, f	8
					- Control group: 35.5 ± 14.7	- Analysis within 2 h			
Fan	China	2015	Retrospective study	320	- Appendicitis group: 45.6 ± 19.6	- Blood obtained time: Not available	- Mean platelet volume (MPV)	a, b, d,e, f	8
					- Control group: 43.0 ± 12.5	- Analysis within 10 min	- Platelet distribution width (PDW)		
Erdem	Turkey	2015	Retrospective study	200	- Appendicitis group: 33.6 ± 12.2	- Blood obtained within 24 h of the diagnosis	Mean platelet volume (MPV)	a, b, c, f	7
					- Control group: 30.8 ± 9.7				
Bozkurt	Turkey	2015	Retrospective study	275	- Complicated appendicitis group: 33	- Not available	- Mean platelet volume (MPV)	a, b, f	7
					- Non-complicated appendicitis group: 31				
					- Control group: 34				
Yardimci	Turkey	2016	Retrospective study	513	- Appendicitis group: 32.4		Mean platelet volume (MPV)	a, b, f	6
					- Control group: 42.7				
Boshnak	Egypt	2017	Prospective non- randomised study	200	- Appendicitis group: 22.36 ± 7.64	- At the time of admission	- Mean platelet volume (MPV)	a, b, c, d, e, f	8
					- Control group: 29.10 ± 16.33	- Analysis within 1 h	- Platelet distribution width (PDW)		
Yigit	Turkey	2019	Retrospective study	322	- Appendicitis group: 32 ± 13	- Blood obtained time: Not available	- Mean platelet volume (MPV)	a, b, d, e, f	8
					- Control group: 32 ± 14	- Analysis with 1 h	- Platelet distribution width (PDW)		
Birick	Turkey	2019	Retrospective study	424	- Complicated appendicitis group: 35.9 ± 16.7	- Not available	- Mean platelet volume (MPV)	a, b, e, f	8
					- Non-complicated appendicitis group: 35.1 ± 13.3				
					- Control group: 32.9 ± 11.7				
Haghi	Iran	2019	Retrospective study	438	- Mean age 26.5 ± 13.9	- Not available	- Mean platelet volume (MPV)	a, b, f	6

Abbreviations: a = age, b = sex, c = time of blood obtained d = time of blood analysis, e = type of anticoagulant in collecting tube, f = pathologic diagnosis of appendicitis, fL = x 10^9^/L.

### 3.1 Mean platelet volume (MPV) and the diagnosis of acute appendicitis

**Fig. 2:**
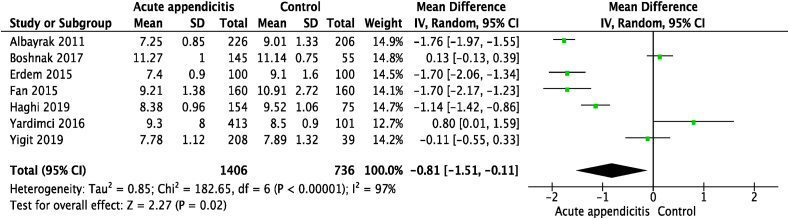
Forest plot of the association between MPV and diagnosis of acute appendicitis.

**Fig. 3:**
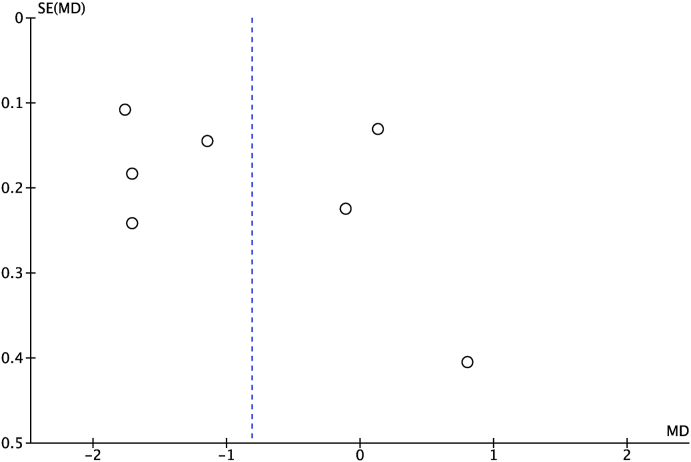
Funnel plot of the association between MPV and diagnosis of acute appendicitis.

Seven studies [[21], [22], [23],^25^,^25^,^27^,^29^] reported an association between MPV and acute appendicitis, with a total of 2142 patients (1406 patients [68%] in the acute appendicitis group and 736 patients [32%] in the control group). Pooled analysis of the seven studies demonstrated that the MPV among patients with acute appendicitis was significantly lower than the control group (OR = −0.81,95% CI = −1.51 to −0.11, P = 0.02). There was significant heterogeneity between studies (I^2^ = 97%, p < 0.00001). A forest plot displaying the association between MPV and diagnosis of acute appendicitis is illustrated in Fig. 2. Thus, the data was analyzed with random effect models. No evidence of publication bias was observed by either Egger's test (P = 0.398) or the rank correlation test (P = 0.652). A funnel plot of the meta-analysis shown symmetrical distribution is illustrated in Fig. 3*.*

### 3.2 Mean platelet volume (MPV) and the prediction of complicated appendicitis

Four studies [24,[26], [27], [28]] reported an association between MPV and complicated appendicitis, with a total of 450 patients (235 patients [52%] in the complicated appendicitis group and 215 patients [48%] in the control group). The pooled analysis demonstrated that MPV appeared to reduce in the complicated appendicitis group, but was not significantly different (OR = −0.13,95% CI = −0.33 to −0.07, P = 0.19). There was no evidence of heterogeneity between the study (I^2^ = 0%, p = 0.57) or publication bias (Egger's test [P = 0.807] or the rank correlation test [P = 0.734]) observed in this analysis.

Additional analysis was conducted with four observational studies [24,26,28,29] that included 931 patients by comparing the MPV in complicated (200 [21%] patients) and non-complicated appendicitis group (731 [79%] patients). There was no significant difference between two patients groups (OR = −0.13,95% CI = −0.30 to −0.04, P = 0.14). Evidence of heterogeneity (I^2^ = 0%, p = 0.14) and publication bias [Egger's test (P = 0.949) or the rank correlation test (P = 1.000)] was not observed.

### 3.3 Platelet distribution width (PDW) and the diagnosis of acute appendicitis and prediction of complication

Three studies [22,26,27] with a total of 767 patients included 513 (67%) patents in the acute appendicitis group and 254 (33%) patients in the control group. The pooled analysis did not demonstrate significant difference in PDW between both patient groups (OR = 1.19,95% CI = −0.59 – 2.97, P = 0.19). Significant heterogeneity between the studies was found (I^2^ = 98%, p < 0.0001). No evidence of publication bias was identified by either Egger's test (P = 0.744) or the rank correlation test (P = 0.602).

Two studies [26,27] investigated the association between PDW and complicated appendicitis which included 153 patients (59 [39%] patients in the complicated appendicitis group and 94 [61%] in the control group). The pooled analysis demonstrates no significant difference in PDW between the complicated appendicitis and control groups (OR = −0.83,95% CI = −2.10 – 0.44, P = 0.20). Significant heterogeneity between the studies was found (I^2^ = 80%, p = 0.02).

The final analysis was conducted from two studies [26,27] which included 353 patients (59 [17%] in the complicated appendicitis group and 294 [83%] in the non-complicated appendicitis group). There was no significant different in PDW in both groups (OR = −1.46, 95% CI = −4.32 – 1.40, P = 0.32). Significant heterogeneity was detected (I^2^ = 96%, p < 0.00001). A summary of the association between platelet indices (MPV and PDW) and the diagnosis of acute appendicitis and prediction of complicated appendicitis is illustrated in Table 2.

**Table 2:** The result of the meta-analyses of the association between platelet indices (MPV and PDW) and the diagnosis of acute appendicitis and prediction of complicated appendicitis.

Comparison	Number of studies	n	Odds Ratio	95% confidential interval	P value	Heterogeneity between the study	Egger's test	Rank-correlation test
Mean platelet volume (MPV) for prediction of complicated appendicitis: comparison with control group	4	450	−0.13	−0.33–−0.07	0.19	I^2^ = 0%, p = 0.57	P = 0.807	P = 0.734
Mean platelet volume (MPV) for prediction of complicated appendicitis: comparison with non-complicated appendicitis group	4	931	−0.13	−0.30–−0.04	0.14	I^2^ = 0%, p = 0.14	P = 0.949	P = 1.000
Platelet distribution width (PDW) for the diagnosis of acute appendicitis	3	767	1.19	−0.59 – 2.97	0.19	I^2^ = 98%, p < 0.0001^a^	P = 0.744	P = 0.602
Platelet distribution width (PDW) or prediction of complicated appendicitis: comparison with control group	2	153	−0.83	−2.10 – 0.44	0.20	I^2^ = 80%, p = 0.02^a^	N/A	N/A
Platelet distribution width (PDW) or prediction of complicated appendicitis: comparison with non-complicated appendicitis group	2	353	−1.46	−4.32 – 1.40	0.32	I^2^ = 96%, p < 0.00001^a^	N/A	N/A

a Statistical significant, N/A = Not available.

## 4 Discussion

Appendectomy is the most common emergency surgical operation worldwide. Normal histopathologic findings of the appendix (negative appendectomy) reported an incidence of 8.47–9.5% which may increase mortality by up to 1.93% in male patients [30]**.** In addition, the most common mistaken diagnoses are typically correlated with young females with gynecological conditions (25–64.3%) [31]. Evaluating patients with the clinical scoring system may lower the negative appendectomy rate to 6.84% [32]. In an attempt to avoid unnecessary appendectomy, serum biomarkers such as C-reactive protein and the Neutrophils-lymphocyte ratio was incorporated with the clinical scoring system to improve diagnosis accuracy.

Platelet indices consist of the mean platelet volume (MVP) and platelet distribution width (PDW) is a biomarker of platelet activation, which is inexpensive, comfortable, and can be rapidly measured by an automated hematology analyzer.

Interleukin-6 (IL-6) is excreted during the inflammatory period in acute abdominal pain such as AA and ovarian torsion which may contribute to platelet sequestration and destruction, leading to activation of megakaryocytes in the bone marrow to release young platelets into circulation. Thus, large platelets should be found in the early inflammatory phase, then the progression into the late phase of sepsis. Meanwhile, small-sized platelets should be detected in CBC analysis and indicate complicated intrabdominal infection. This finding may lead to low sensitivity of MPV and PDW in the diagnosis of AA in some previous studies [33,34].

The meta-analysis results investigated whether MVP and PDW are biomarkers for the diagnosis of acute appendicitis and the prediction of complicated appendicitis. The results demonstrate that MPV is significantly lower among acute appendicitis groups compared to control groups. Similar to Ceylan et al. the MPV value significantly decreased in non-complicated appendicitis subjects with a cutoff level of 9.9500 × 10^9^ L, in which sensitivity and specificity were 59.0% and 59.5%, respectively [35]. Shen et al reported the association between decreasing MPV value which was associated with acute appendicitis (SMD − 0.34; 95% CI − 0.56 to − 0.12; *P* = 0.003). However, the level of MPV also decreased in non-complicated appendicitis patients, but the interpretation of this finding may be limited by the positive results from small sample studies [36]**.** This evidence supports the efficacy of MPV as a biomarker for the diagnosis of acute appendicitis.

Nevertheless, the results of the present study fail to demonstrate the diagnostic value of MPV for the prediction of complicated appendicitis. There appears to be lower MPV values among complicated appendicitis groups, but not at a significant degree of difference from the control and non-complicated appendicitis groups. This finding could be explained by: 1) The severity of inflammation and sepsis; 2) Timing of blood samples obtained; and 3) Only three of the nine included studies [23,26,29] excluded patients with a history of blood transfusion and use of anti-coagulants or non-steroidal anti-inflammatory drugs (NSAIDS) which can affect platelet morphology and function.

During the early phase of inflammation, large platelets are released from the bone marrow. The progression of the high-grade inflammatory activity resulted in the large platelets get sequestrated and destroyed in the inflammatory zone, and small platelets become dominant.

Furthermore, MPV may enlarge during the sepsis period due to platelet activation instead of increasing the production of young platelets from megakaryocytes which takes time [26,37,38].

In contrast to the association between MPV and acute appendicitis, the pooled analysis of recent literature demonstrates that PDW has no diagnostic value in acute appendicitis and is unable to predicts its complication. Fan et al. described that increasing PDW at the cut-off level 15.1 × 10^9^/L should be a valuable diagnostic marker of acute appendicitis with a 76.3% sensitivity and 93.1% specificity [22]. The difference between studies can be described as: 1) Patient comorbidities - chronic disease that may affect platelet morphology such as obesity, hypertension, smoking, and hyperlipidemia; and 2) Pre-analytical factors - anticoagulant in collecting tubes used for blood samplings may affect the platelet morphology (EDTA-induced platelets swelling, citrate induced platelet shrinkage) may lead to unreliable outcomes [39,40].

The results of the present study were somewhat complicated since significant heterogeneity was identified in the meta-analysis of the association between MPV and PDW with the diagnosis of acute appendicitis and PDW with complicated appendicitis. The heterogeneity observed in the meta-analysis can be explained by: 1) Differentiation in the patient exclusion criteria for each study; 2) Timing of blood samples obtained varied from patients presenting time to within 24 h after diagnosis; 3) Waiting time for blood analysis varied from 10 min to 2 h; and 4) The disparity of hematology analyzers and the reference values of each study. Additionally, the data was adjusted to mimic the heterogeneity in the analysis of MPV and complicated appendicitis and adopted a random-effects model to calculate the OR in order to compensate this effect for a more conservative result.

The major limitations of the present study include several potential sources of publication bias, for instance the inclusion of only English language publications, studies that indicate continuous variable outcomes may lead to missing relevant articles, and only observational retrospective studies were considered. Finally, most of the patients in this analysis were from Asian populations and does not represent a global clinicopathological correlation between platelet indices and acute appendicitis. Nonetheless, evidence of publication bias was not observed from the Egger's linear regression test and rank correlation test results.

## 5 Conclusion

This study demonstrates that lower MPV values can have a significant role in the diagnosis of acute appendicitis, but failed to determine severity. The researchers suggest the use of MPV as a biomarker along with the clinical scoring system in patients with suspected acute appendicitis for greater diagnostic accuracy. Nevertheless, PDW was not found to be useful as a diagnostic marker and prediction of complicated appendicitis.

## Provenance and peer review

Not commissioned, externally peer-reviewed.

## Presentation at a meeting

No.

## Author contribution

Tullavardhana Thawatchai: Concepts, Design, Definition of intellectual content, Literature search, Clinical studies, Data analysis, Statistical analysis, Manuscript review, Guarantor, Sanguanlosit Sarat: Design, Definition of intellectual content, Literature search, Data acquisition, Statistical analysis, Manuscript preparation, Manuscript review, Guarantor, Chartkitchareon Anuwat: Definition of intellectual content, Literature search, Data acquisition, Data analysis, Manuscript editing.

## Sources of funding

The study received grant supports from 10.13039/100009570Srinakharinwirot University, Thailand

## Ethical approval

This study is a systematic review and meta-analysis: no need for ethical approval.

## Registration of research studies

Name of the registry: THAWATCHAI TULLAVARDHANA.

Unique Identifying number or registration ID: reviewregistry1131.

Hyperlink to your specific registration (must be publicly accessible and will be checked): https://www.researchregistry.com/browse-the-registry#registryofsystematicreviewsmeta-analyses/registryofsystematicreviewsmeta-analysesdetails/60744d3e348b8b001c745bfa/

## Guarantor

Associated Professor Thawatchai Tullavardhana.

## Declaration of competing interest

The authors declare no conflicts of interest.
